# Akinetes From Late Paleoproterozoic Salkhan Limestone (>1600 Ma) of India: A Proxy for Understanding Life in Extreme Conditions

**DOI:** 10.3389/fmicb.2019.00397

**Published:** 2019-03-12

**Authors:** Mukund Sharma, Bandana Shukla

**Affiliations:** Birbal Sahni Institute of Palaeosciences, Lucknow, India

**Keywords:** akinetes, authigenic doubly-terminated quartz crystal, chert, extreme environment, fan-fabrics, Salkhan Limestone, India

## Abstract

Isolated elongate spore-like cells present in the >1600 Ma-old Salkhan Limestone of the Semri Group, Vindhayan Supergroup, India are considered akinetes of the heterocystous cyanobacteria. Small to large size, and young (single walled) to mature (double walled) akinetes – namely, *Archaeoellipsoides bactroformis, A. conjuctivus, A. dolichos, A. elongatus*, *A. grandis, A. major* and *A. minor* – found in the stromatolitic and bedded cherts have been reported in the present paper. Their role in understanding extreme environmental conditions is a subject matter of this paper. Additionally, the occurrence of doubly-terminated quartz crystals and fan-fabrics in the Salkhan Limestone indicates adverse conditions for the survival of life forms. The depositional environment of the Salkhan Limestone, Vindhyan Supergroup is suggested to be shallow marine intertidal with pulses of the intermittent hypersaline regime during which akinetes, closely resembling those of extant Nostocaceans, were formed by cyanobacteria for survival in the extreme conditions.

## Introduction

The origin of varied life forms during the Proterozoic Eon (2500-541 Ma) and their biology, ecology, and preservation in the different depositional environments are a subject of great interest to Precambrian paleobiologists. Some of these issues and associated processes are being investigated to understand life forms in extreme conditions as proxies for and signatures of life on other planetary bodies. Heterocystous fossil cyanobacteria and their various degradational states are potentially useful for inferring the prevailing extreme environmental conditions in the geological past. Documentation of silicified microfossils in the Precambrian Gunflint chert of Canada is considered to be the beginning of the Precambrian paleobiology (Tyler and Barghoorn, 1954). Initial studies conducted on Precambrian successions across the globe demonstrated that silicified microfossils entombed in the chert are conspicuously dominated by filamentous and coccoid microfossils, most of which are comparable to modern cyanobacteria (Barghoorn and Tyler, 1965; Schopf, 1968; Schopf and Blacic, 1971; Knoll, 1982, 1984, see Sergeev et al., 2012 for synthesis of such studies). These studies are, however, primarily based on a taxonomic approach; therefore, the evolutionary inferences drawn from these studies are few. Subsequently, paleoenvironmental interpretations based on microfossil assemblages were also recorded (Golubic, 1973, 1976; Golubic and Hofmann, 1976; Hofmann, 1976; Golubic and Barghoorn, 1977; Golubic and Campbell, 1979; Horodyski and Donaldson, 1980, 1983; Knoll, 1985a; Green et al., 1988, 1989; Knoll and Golubic, 1992; Golubic and Seong-Joo, 1999; Sharma and Sergeev, 2004; Sharma, 2006a,b).

The Precambrian period is generally known as the most crucial time in geological history for understanding the growth and evolution of surviving life forms, especially those in extreme environmental conditions (Young, 2013). Studies conducted on the Salkhan Limestone of the Semri Group in India demonstrated the diversity of life forms thriving during the deposition of the Salkhan Limestone and suggested how environmental conditions prevailed during the late Paleoproterozoic period (Kumar, 1978a,b; McMenamin et al., 1983; Venkatachala et al., 1990; Kumar and Srivastava, 1992a,b, 1995; Sharma and Sergeev, 2004; Prasad et al., 2005; Srivastava, 2005; Sharma, 2006a,b; Sergeev et al., 2008; Srivastava and Tewari, 2011). However, the relation of these life forms to extreme environmental conditions is largely unknown and requires a thorough explanation.

Well-preserved cyanobacterial remains in the form of microfossils in the chert samples of the Salkhan Limestone, Vindhyan Supergroup have been recorded (Kumar and Srivastava, 1995). In extant aquatic environments, both marine and freshwater, some cyanobacteria develop two specialized types of cells: (i) heterocysts, which help fix atmospheric nitrogen and make cyanobacteria important for N_2_-fixation, and (ii) akinetes, which act as a survival strategy and become dormant cells that form under adverse conditions, re-germinating to form new cyanobacterial cells upon the onset of more favorable conditions (Adams and Duggan, 1999; Pinto et al., 2016). As such, akinetes are huge, resting-state cyanobacterial cells that are surrounded by a thick cell wall and a multilayered extracellular envelope (Nichols and Adams, 1982; Herdman, 1987, 1988). Therefore, the occurrence of akinetes in any depositional environment can potentially be used to interpret adverse/extreme environmental conditions in the geological past (Tomitani et al., 2006). Studies conducted on the microfossil assemblage documented from the chert samples of the Salkhan Limestone of the Newari locality were characterized by the presence of different types of akinetes (Srivastava, 2005; Sharma, 2006b); however, the occurrences of these akinetes and their relation to extreme environmental conditions during the late Paleoproterozoic period is yet to be explained thoroughly. We present here the data on akinetes recorded in the thin sections of the chert samples of the Salkhan Limestone, Vindhyan Supergroup, India. Additional data on the depositional environment, associated minerals, and petrological studies on the Salkhan Limestone helped to understand prevailing extreme environmental conditions and the strategies adopted by the akinetes for their survival. The conspicuous occurrence of authigenic doubly-terminated quartz crystals in the surroundings of akinete-bearing cherts is intriguing. The authigenic doubly-terminated quartz crystals are widely reported from different geological settings worldwide and considered to form under evaporitic conditions (Tarr and Lonsdale, 1929; Folk, 1952; Grimm, 1962; Wilson, 1966; Zenger, 1976; Ulmer-Scholle et al., 1993; Chafetz and Zhang, 1998; Albright and Lueth, 2003).

## Study Area

**FIGURE 1:**
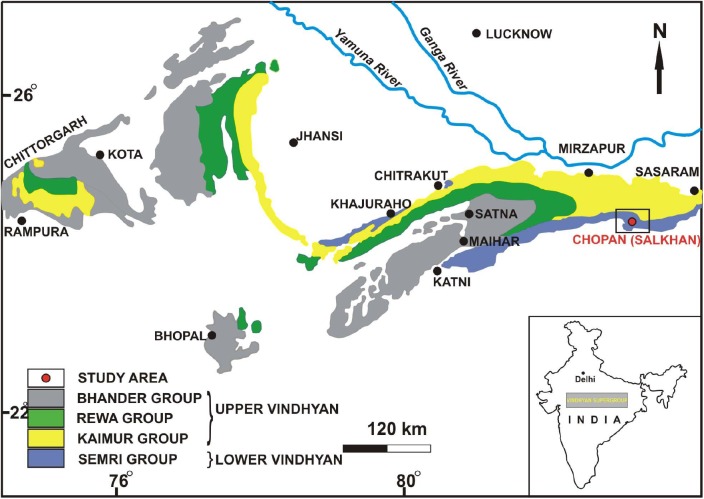
Generalized geological map of Vindhyan Basin, inset map of India showing position of the Vindhyan Supergroup (after Krishnan and Swaminath, 1959).

**FIGURE 2:**
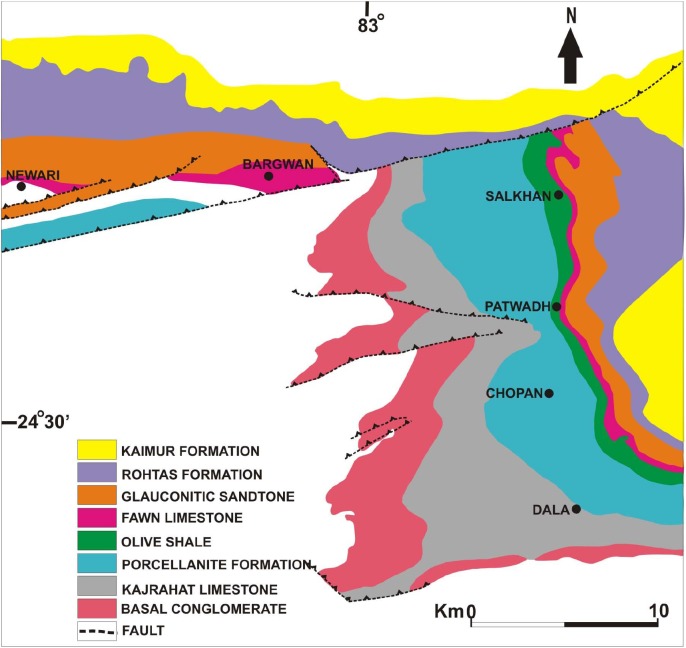
Geological map showing lithostratigraphic units of the Son Valley Section (after Auden, 1933).

The Vindhyan Supergroup is situated in central India and is characterizes by a 4500–5000-m-thick sedimentary succession belonging to the Proterozoic Era. This mostly undeformed and unmetamorphosed thick succession represents a sequence of sandstone, shale, limestone, and dolomite with minor conglomerate as well as volcanoclastic rocks (Auden, 1933). The Vindhyan Supergroup is exposed over a large area from Bihar (Sasaram) in the east to Chittorgarh, Rajasthan in the west, and Dholpur, Rajasthan in the north to Hoshangabad (Madhya Pradesh) in the southwest (Figure 1). The outcrops of the Vindhyan Supergroup are present between the Archaean Aravalli-Bundelkhand province (to the north and east) and the Cretaceous Deccan Traps (to the south and bounded by the Great Boundary Fault to the west) (Mazumder et al., 2000). Lithostratigraphically, the Vindhyan Supergroup represents four groups, namely the Semri Group, the Kaimur Group, the Rewa Group, and the Bhander Group. The Semri Group is considered as the Lower Vindhyans, whereas the Kaimur, Rewa, and Bhander Groups together constitute the Upper Vindhyans. The Kheinjua Subgroup, part of the Semri Group, is lithostratigraphically divided into the three formations, namely the Olive Shale, the Salkhan Limestone, and the Glauconitic Sandstone (Figure 2). In a broader context, the Vindhyan Basin is further divided into two parts which are the eastern part and the western part. The eastern part is known as the Son Valley Section, whereas the western part is the Chambal Valley Section. The best exposure of the Semri Group is in the Son Valley area, from east to west, Rohtas district, Bihar, Sonbhadra district, Uttar Pradesh (Figure 2), and Satna district, Madhya Pradesh. The Salkhan Limestone is a ∼90-m-thick unit of fawn and dark grayish color, mainly constituted of dolomitic, siliceous limestone, and chert bands. The Vindhyan basin is believed to be deposited under the shallow marine environment (Chakraborty et al., 2012 and references therein). More specifically, the depositional environment of the Salkhan Limestone of the Semri Group has been proposed to be intertidal to a supratidal environment based on the microbial assemblages (Sharma, 2006a).

## Materials and Methods

**FIGURE 3:**
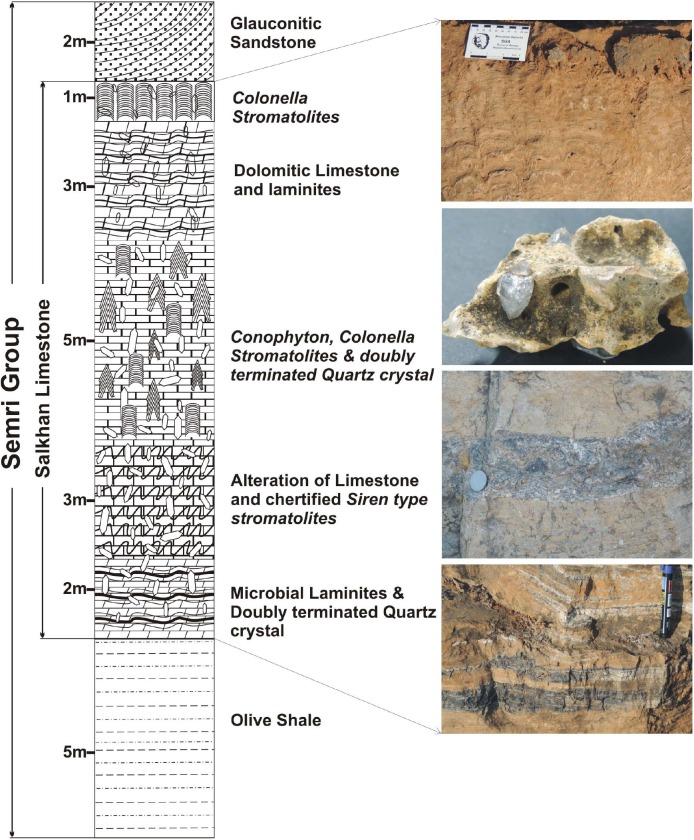
Litho-column showing the Salkhan Limestone section exposed in the Newari in Sonbhadra district, Uttar Pradesh, India.

Akinete-yielding chert samples were collected from the various localities of the Salkhan Limestone of the Semri Group (exposed in the Sonbhadra and Rohtas districts of India) whereas doubly-terminated quartz crystals were collected from the Newari area (Figure 3). The thin sections of the chert samples were studied for the diversity of microbial life, including the akinetes (*Archaeoellipsoides*). Akinetes and fan-fabrics were examined and photographed under transmitted light using a Nikon Eclipse 80*i* Microscope. All the photographs included in different plates were taken using the NIS Element F 3.0 program of the microscope. It should be noted that this photo-imaging technique sometimes shows duplicating images of the content; therefore, all the focused zones were sutured using the COREL Draw 12 program to make completely focused specimens. The size measurements of akinetes were carried out using an eyepiece micrometer. Two main techniques, namely maceration and thin sections, were used to recover and study the microfossil assemblages in chert samples. Slide No. (S.No. BSIP-10907, 14993, 14994, 14997-14999, 15107, 15723-15741) and England Finder co-ordinates (EF) are also provided for all the documented akinetes and listed in Supplementary Data. All the slides are deposited in the repository of the Birbal Sahni Institute of Palaeosciences, Lucknow, India under statement nos. 825, 1386, and 1431. The doubly-terminated quartz crystals were observed visually and photographed.

## Results

### Akinetes

**FIGURE 4:**
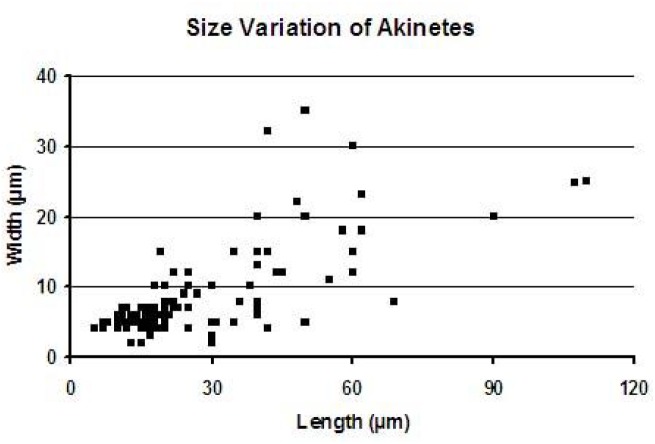
Size variation diagram of akinetes occurring in the thin section of cherts from the Salkhan Limestone.

We present here the results of observations on the akinetes obtained from the chert samples of the Vindhyan Supergroup, India. The size measurements of akinetes revealed a wide range of size with length ranging from 2 to 110 μm and width ranging from 2 to 35 μm (Figure 4). A detailed account of *Archaeoellipsoides* reported from the Salkhan chert samples is tabulated in Table 1. The taxonomic characterization of the akinetes is as follows:

**Table 1:** A list of recorded akinetes in the present study showing size range from the Salkhan Limestone.

S. No	Name of Species	Size in μm		References
		Length	width	
1	*Archaeoellipsoides bactroformis*	50–70	5–15	Sergeev et al., 1995
2	***Archaeoellipsoides conjuctivus***	44–110	12–25	Zhang, 1985
3	*Archaeoellipsoides dolichos*	10–42	2–7	(Zhang, 1985), comb. Sergeev and Knoll, 1995 (in Sergeev et al., 1995)
4	*Archaeoellipsoides elongatus*	18–40	4–8	(Golovenok and Belova, 1984), Sergeev et al., 1995
5	*^∗^Archaeoellipsoides grandis*	35–107	15–24	Horodyski and Donaldson, 1980 emend. Golovenok and Belova, 1984, emend. Sergeev and Knoll, 1995 (in Sergeev et al., 1995)
6	*Archaeoellipsoides major*	18–60	5–30	Golovenok and Belova, 1984, emend. Sergeev and Knoll, 1995 (in Sergeev et al., 1995)
7	*Archaeoellipsoides minor*	2–24	2–10	Sergeev and Knoll, 1995 (in Sergeev et al., 1995)


*The species denoted in bold has been recorded for the first time from the Proterozoic successions of India; the species marked with an asterisk has been recorded for the first time from the Salkhan Limestone.*

### Taxonomy

**Kingdom :** Eubacteria Woese and Fox, 1977

**Phylum :** Cyanobacteria Stainer et al., 1978

**Class :** Coccogoneae Thuret, 1875

**Order :** Nostocales Geitler, 1925

**Family :** Nostocaceae Kützing, 1843

**Genus :** *Archaeoellipsoides* Horodyski and Donaldson, 1980 emend. Sergeev et al., 1995.

**FIGURE 5:**
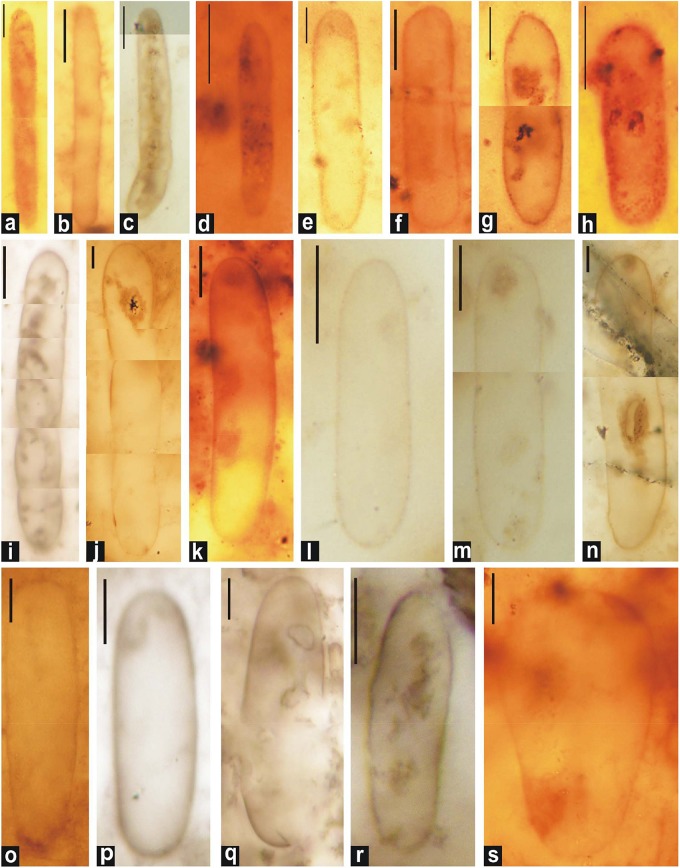
Photomicrographs of single-walled akinetes in petrographic thin sections of chert. **(c, e,** and **l)** *Archaeoellipsoides bactroformis*; **(i,o)** *A*. *conjuctivus*; **(b,d)** *A*. *dolichos*; **(a,f)** *A*. *elongatus*; **(j,k,n,q,** and **s)** *A*. *grandis*; **(g,m,p,** and **r)** *A*. *major*; **(h)** *A*. *minor*. Scale bar = 10 μm.

**FIGURE 6:**
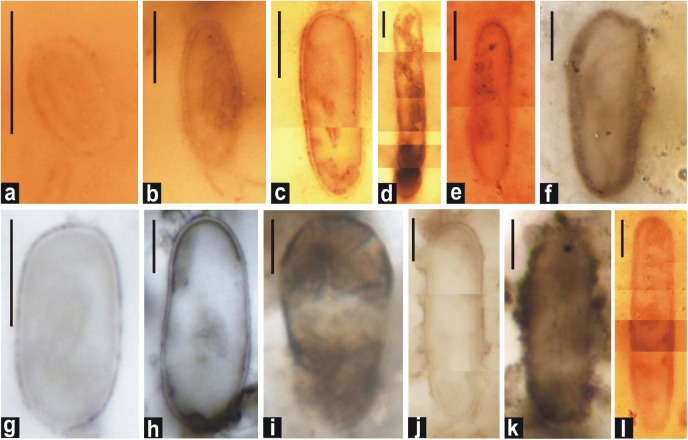
Photomicrographs of double-walled akinetes formed in petrographic thin sections of black chert. **(d,f,h–j,** and **l)**: *A*. *bactroformis*; **(c,k)** *A*. *major*; **(a,b,e,** and **g)**: *A*. *minor*. Scale bar = 10 μm.

**Type Species :** *Archaeoellipsoides bactroformis* Sergeev et al., 1995 (Figure 5c,e,l, 6d,f,h–j,l).

***Description:*** Solitary, single-, and double-layered ellipsoids with rounded ends, cell walls of ellipsoids medium to coarse-grained; empty or containing amorphous organic matter, elongated dark bodies. Length of ellipsoids 50–70 μm, width 5–15 μm, length width (L/W) ratio 4–10.

***Remarks:*** The Salkhan ellipsoidal microfossils are small in cross-section and comparable with the Billyakh and Shorikha microfossils. There is no evidence of binary cell divisions. The close similarity in L/W ratio of these specimens with *A. bactroformis* and the wide variation in length allow these to be considered as *A.* aff. *bactroformis*.

***Age and Locality:*** Paleo- to Mesoproterozoic; Nauhatta (Bihar), Newari and Jata Shankar Pahari (Sonbhadra district, Uttar Pradesh) localities of the Salkhan Limestone, India.

**FIGURE 7:**
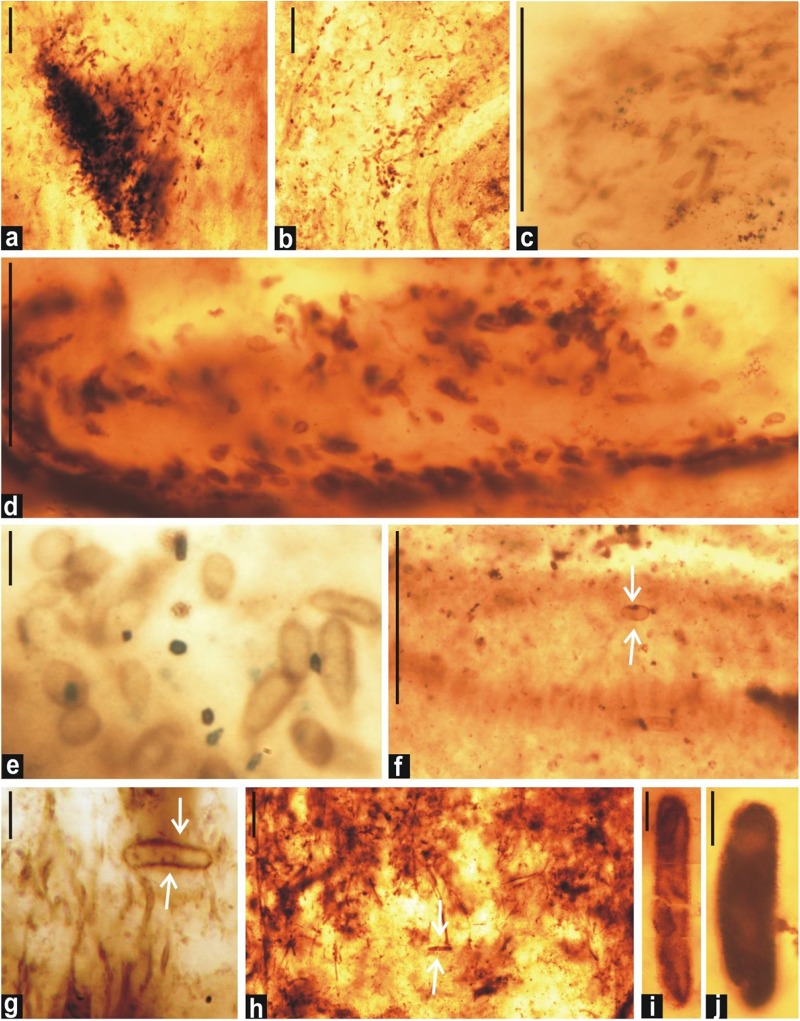
Distribution pattern of small-sized akinetes observed in petrographic thin sections of the black chert of the Salkhan Limestone. Assemblage showing the cluster and random distribution of *Archaeoellipsoides* **(a–e)**. Note the occurrence of solitary individual of *Archaeoellipsoides* (marked by arrow) entangled in the radial fan-fabric **(f)**. Individual *Archaeoellipsoides* (marked by arrow) are shown along with the filamentous cyanobacteria **(g,h)**. *Archaeoellipsoides* shown in **(a–g)** represent *A.* *minor*, **(h,i)** *A. conjuctivus*, and **(j)** *A. elongatus*. Specimens **a–d, f**, and **h** are photographed at low magnification and scale bars represent 100 μm whereas scale bars in **e,g,i**, and **j** represent 10 μm.

***Archaeoellipsoides conjuctivus*** Zhang (1985) (Figure 5i,o, 7h,i).

***Description:*** Ellipsoids solitary, single-layered, and slightly curved with flattened ends; basically empty but sometimes containing blebs of irregular micron-sized granules attached to the inner side of the ellipsoid; walls of ellipsoids medium- or fine-grained about 0.5 μm thick. The range of the length of ellipsoids is 44–110 μm, width 12–25 μm, and the length-width ratio is 1.4 to 4.5.

***Remarks*:** Zhang (1985) diagnosed *A. conjunctivus* as chains of ellipsoidal bodies. That distinction is retained here but is purely formal; most Kotuikan and Yusmastakh akinetes were possibly arranged in chains originally. On the other hand, the modern cyanobacterial species *Aulosira* forms a chain of akinetes which is surrounded by a common sheath (Elenkin, 1938, p. 872) similar to *A. conjunctivus*.

***Age and Locality:*** Paleo- to Mesoproterozoic; Nauhatta (Bihar), Newari, and Jata Shankar Pahari (Sonbhadra district, Uttar Pradesh) localities of the Salkhan Limestone, India.

***Archaeoellipsoides dolichos*** (Zhang, 1985), comb. Sergeev and Knoll, 1995 (in Sergeev et al., 1995) (Figure 5b,d).

***Description:*** Single-layered solitary, straight or gently curved rod-like ellipsoids with rounded ends; vesicles empty and 10–42 μm long but only 2–7 μm wide, length-width ratio 2 to 15.

***Remarks:*** *A. dolichos* differs from other species of *Archaeoellipsoides* by its small cross-sectional diameter and high length-width ratio.

***Age and Locality*:** Paleo to Mesoproterozoic; Newari (Sonbhadra district, Uttar Pradesh) locality of the Salkhan Limestone, India.

***Archaeoellipsoides elongatus*** (Golovenok and Belova, 1984), comb. Sergeev and Knoll, 1995 (in Sergeev et al., 1995) (Figure 5a,f, 7j).

***Description:*** Solitary, single-layered vesicles with rounded ends; vesicles generally empty, but may contain sparse blebs of amorphous organic matter. Ellipsoidal vesicles 18–40 μm long and 4–8 μm wide, length-width ratio 2.5 to 6.6.

***Remarks*:** These elongated ellipsoidal microfossils were described by Golovenok and Belova (1984) as a distinct species of *Eosynechococcus*. Subsequently, Zhang (1985) described similar fossils as *Bactrophycus oblongus* from the Wumishan Formation. Some elongated bodies from the Dismal Lakes Group referred to by Horodyski and Donaldson as *Oscillatoriopsis curta* are probably remnants of akinetes comparable to *Archaeoellipsoides elongatus* (Horodyski and Donaldson, 1980, Figures 13A,B,G and Horodyski and Donaldson, 1983, Figure 5AA).

***Age and Locality:*** Paleo to Mesoproterozoic; Newari (Sonbhadra district, Uttar Pradesh) locality of the Salkhan Limestone, India.

***Archaeoellipsoides grandis*** Horodyski and Donaldson, 1980 emend. Golovenok and Belova, 1984, emend. Sergeev and Knoll, 1995 (in Sergeev et al., 1995) (Figure 5j,k,n,q,s).

***Description:*** Solitary, single- and double-layered ellipsoids with rounded ends; ellipsoids usually empty and slightly curved; while blebs of amorphous organic matter found inside ellipsoids. Vesicle wall medium to coarse-grained, ellipsoids 35–107 μm long and 15–24 μm wide; length-width ratio of 2 to 4.3.

***Remarks:*** Sergeev (1992) described ellipsoidal bodies as *A. longus* from the Chichkan Formation of South Kazakhstan, which was in accordance with the formal classification of genus *Archaeoellipsoides* (Sergeev et al., 1995), and should be transferred to *A. grandis*.

***Age and Locality:*** Paleo- to Mesoproterozoic; Nauhatta (Bihar) and Jata Shankar Pahari (Sonbhadra district, Uttar Pradesh) localities of the Salkhan Limestone, India.

***Archaeoellipsoides major*** Golovenok and Belova, 1984, comb. Sergeev and Knoll, 1995 (in Sergeev et al., 1995) (Figure 5g,m,p,r, 6c,k).

***Description:*** Solitary, double-layered ellipsoids with rounded ends; ellipsoids basically empty, length 18–60 μm and width 5–30 μm, length-width ratio of 1.2 to 4.8, defined by translucent medium-grained walls, 1.0 to 1.5 μm thick. Vesicles tend to be aggregated in loose clusters or in short unconnected chain-like groups.

***Remarks:*** Like the other species of *Archaeoellipsoides*, *A. major* is also distinguished by its characteristic range of diameters.

***Age and Locality:*** Paleo- to Mesoproterozoic; Nauhatta (Bihar), Newari, and Jata Shankar Pahari (Sonbhadra district, Uttar Pradesh) localities of the Salkhan Limestone, India.

***Archaeoellipsoides minor*** Sergeev and Knoll, 1995 (in Sergeev et al., 1995) (Figure 5h, 6a,b,e,g, 7a–g).

***Description:*** Mostly found in the cluster, sometimes solitary, single and double layered ellipsoids with rounded and tapering ends; mainly empty vesicles sometimes containing amorphous organic matter. Vesicle wall medium to coarse-grained, ellipsoids are 5–24 μm long and 2–10 μm wide. Length-width ratio is 1.2 to 7.5.

***Remarks:*** Sergeev et al. (1995) reassigned the ellipsoids described by Golovenok and Belova (1984) as *Eosynechococcus grandis* to *Archaeoellipsoides minor* and also the specimens described by Horodyski and Donaldson (1980) as a plausible chain of *A. minor*. They compared the specimens to akinetes of living *Anabaena flos-aquae* that commonly occur as clumps. The Salkhan population of *A. minor* is smaller in size.

***Age and Locality:*** Paleo- to Mesoproterozoic; Nauhatta (Bihar), Newari, and Bargwan (Sonbhadra district, Uttar Pradesh) localities of the Salkhan Limestone, India.

### Authigenic Doubly-Terminated Quartz Crystals and Fan-Fabrics

**FIGURE 8:**
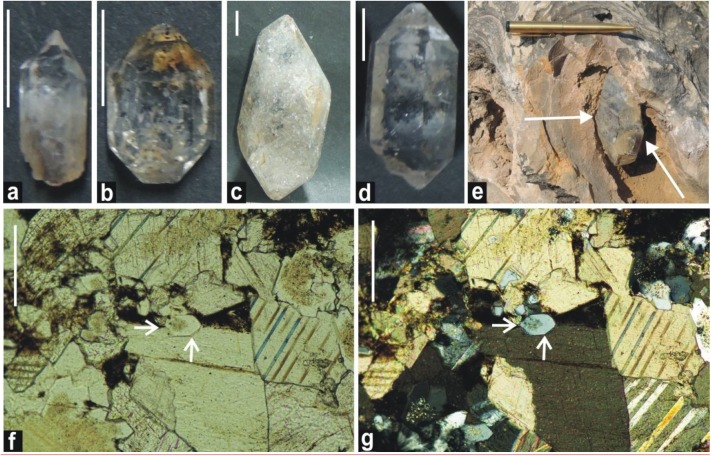
Variety of doubly-terminated quartz crystals from the Salkhan Limestone recorded at Newari locality. Hand specimens of colorless, prismatic, white, and smoky quartz crystals are shown **(a–d)** and scale bars for the same are 1 cm. Field photograph shows the relationship of quartz crystal with associated stromatolite, length of pen is 13.2 cm **(e)**. Doubly-terminated quartz crystal in petrographic thin sections under the plane and cross polarized light indicated by white arrow **(f,g)**.

Doubly-terminated quartz crystals and fan-fabrics have been recorded in the Salkhan Limestone of the Vindhyan Supergroup. More than a hundred such crystals have been collected from the field, and a few hand specimens of massive limestone that contain quartz crystals are available for study. All the quartz crystals are euhedral. Their sizes range from a few millimeters to 15 centimeters. These quartz crystals are associated with the dolostone bed of the Salkhan Limestone and are found embedded in the stromatolitic chert unit of the Salkhan Limestone. These are prominently found near the Newari and Bargawan localities in Sonbhadra district. Petrographic thin sections show the presence of early diagenetic chert replacing the micritic part of the limestone. Some of the petrological thin sections of the chert also show the presence of micron-sized quartz crystals. No preferred orientation of these crystals was noted, however, some of them were found cut across the stromatolitic laminae. However, most of these crystals were regular in shape a few were found to be incomplete at one end. Intergrown crystals and twinning were rare. Prismatic faces were smooth in most of the crystals, with a few pits on the rhombohedral faces. On the color parameter, small quartz crystals were mostly clear and colorless. A few were smoky in color, and the larger crystals had some black spots (Figure 8). Doubly-terminated complete crystals were considered as authigenic to the Salkhan Limestone.

**FIGURE 9:**
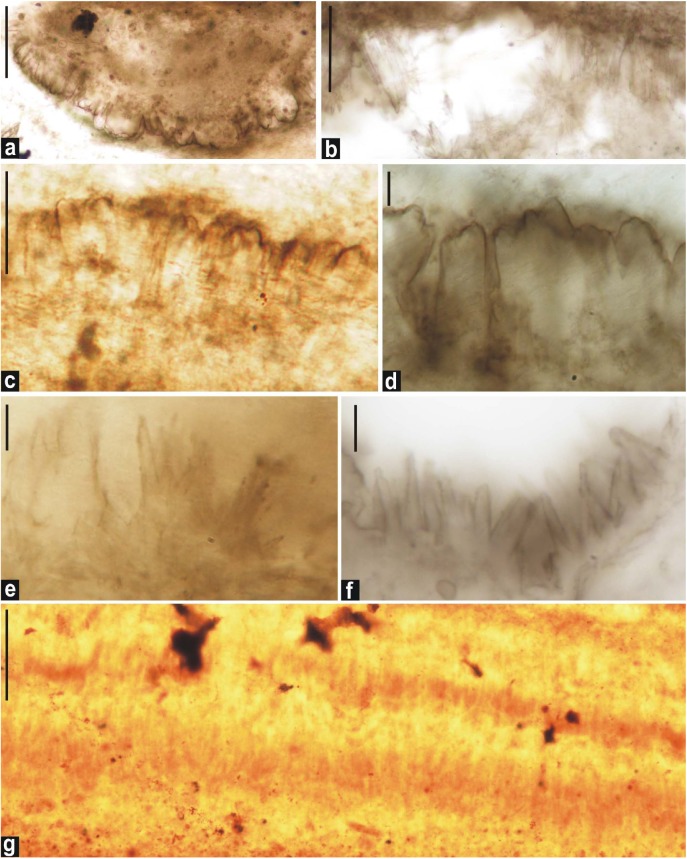
Fan-fabrics from the thin sections of chert from the Salkhan Limestone. **(a)**: Distribution of coccoid cyanobacterial microfossils over fan-shaped blades of aragonite crystals pseudomorphs; **(b–f)**: gradual enlargements of the blade shaped crystal pseudomoprhs of aragonite; **(g)**: akinetes entrapped in crystal pseudomorphs. Scale bars = **a,b,g** = 100 μm and **c–f** = 10 μm.

The observed radial fan-fabrics in the thin sections, in which numerous microfossils were found embedded, are very peculiar. The size of fan-fabrics ranged from 2 to 10 μm (Figure 9).

## Discussion

We reconciled our data on the akinetes (size variations and taxonomic diversity) vis-à-vis authigenic doubly-terminated quartz crystals and demonstrated that the depositional environment of the Salkhan Limestone experienced hypersaline conditions intermittently during the late Paleoproterozoic to early Mesoproterozoic (∼1600 Ma), which forced life forms to adopt survival strategies for the extreme environmental conditions of heat and dryness.

### Formation of Akinetes in the Modern Environment

The heterocystous cyanobacteria usually form akinetes during unfavorable conditions, for which various factors are noted in the modern environment such as light, temperature, nutrient availability, and salinity. Akinetes can remain dormant for years and germinate to develop as new filaments when conditions become favorable (Nichols and Adams, 1982; Herdman, 1987). Both light limitation (Fay, 1969; Sutherland et al., 1979; Nichols et al., 1980; Fay et al., 1984) and increased light intensity (Rother and Fay, 1977; Moore et al., 2005) are important factors that influence the formation of akinetes in some cyanobacterial species. Likewise, nutrient availability (Fisher and Wolk, 1976; Sinclair and Whitton, 1977; Sutherland et al., 1979; Nichols and Adams, 1982; Fay et al., 1984; Rao et al., 1987; Sarma and Khattar, 1993; van Dok and Hart, 1996; Sukenik et al., 2007) and temperature (Li et al., 1997; Moore et al., 2005) also play an important role for the formation of akinetes in certain species, but akinetes do not survive in long exposures to high temperature (Oren, 2014). Salinity is also an important factor for the formation of akinetes, both in freshwater and marine species of cyanobacteria, and akinetes are readily formed when salinity levels are low or high compared to the required growth level (Pandey and Talpasayi, 1981; Huber, 1985; Baker and Bellifemine, 2000). The occurrence of akinetes in shales may indicate such conditions (noted above) in planktonic cyanobacteria in water bodies of normal chemistry. In such conditions, the akinetes form at the end of the optimal vegetation period, sink and accumulate in the sediment, where they survive over winter. They germinate in the next spring and return to the plankton stage during the isothermic turnovers.

### Fossil Records of Akinetes

The recorded forms of akinetes from the Salkhan Limestone belong to the genus *Archaeoellipsoides* Horodyski and Donaldson, 1980. The occurrence of these isolated rod-shaped and large ellipsoidal vesicles (up to 100 μm) was recorded from the Dismal Lakes Group in Canada (Horodyski and Donaldson, 1980). A similar type of assemblage in the chert samples of the Mesoproterozoic Billyakh Group of the Western Anabar region, Northern Siberia was, however, assigned as *Synechococcus*-like cells (Golovenok and Belova, 1981, 1984). The ellipsoidal akinetes preserved in shales were retrieved either through the maceration technique and assigned as *Brevitrichoides* species (Jankauskas et al., 1989) or those found in the thin sections were regarded as *Archaeoellipsoides*. However, some akinetes from the chert were also assigned as *Brevitrichoides*, for instance from the Paleoproterozoic Epworth Group, Canada (Hofmann and Grotzinger, 1985). Heterocysts and akinetes are reported from Paleoproterozoic rocks of Franceville Group, Canada (Amard and Bertrand-Sarfati, 1997), Odjick and Rocknest Formations, and Epworth Group, northwestern Canada (Hofmann and Grotzinger, 1985).

Akinete populations have also been reported from many peritidal carbonates of Mesoproterozoic age, including the Gaoyuzhang and Wumishan Formations, China (Zhang, 1981, 1985; Zhang, 1982; Zhang and Li, 1985; Cao, 1992; Seong-Joo and Golubic, 1999), the Uluksan Group of Baffin Island, Canada (Hofmann and Jackson, 1991), the Kheinjua Formation, India (McMenamin et al., 1983; Kumar and Srivastava, 1995; Srivastava, 2005; Sharma, 2006b), the Deoban Limestone Formation, Garhwal Lesser Himalaya, India (Srivastava and Kumar, 2003), the Sukhaya Tunguska Formation, Turukhansk Uplift, Siberia (Sergeev et al., 1997; Sergeev, 1997, 1999), the Kotuikan and Yusmastakh Formations, Anabar Uplift, north-eastern Siberia (Golubic et al., 1995; Sergeev et al., 1995), and the Debengda Formation, northern Siberia (Sergeev et al., 1994). The akinetes of genera *Archaeoellipsoides* declined after the Mesoproterozoic; the two Neoproterozoic occurrences described to date come from the Chickhan Formation of southern Kazakhstan (Sergeev, 1989, 1992) and the Shorikha Formation, Turukhansk Uplift, Siberia (Sergeev, 2001).

Expanding investigations for the search of microbial remains revealed the presence of a large population of akinetes from the Salkhan Limestone. Sharma and Sergeev (2004) recorded varied precipitate patterns and entrapped *Archaeoellipsoides* in the cherts of the Salkhan Limestone from the Nauhatta area, Rohtas district, Bihar. Srivastava (2005) and Sharma (2006b) recorded *Archaeoellipsoides* in the thin sections of the chert from the Salkhan Limestone. They suggested that these fossil akinetes are similar to those of *Anabaena* species. Besides akinetes, there are also few reports of heterocystous cyanobacteria from Proterozoic sedimentary rocks. Licari and Cloud (1968); Cloud (1976), and Awramik and Barghoorn (1977) interpreted Gunflint fossil *Gunflintia* as heterocysts or akinetes but Knoll (1986) considered them as diagenetic embolisms or differential shrinkage within the filamentous sheaths. Schopf (1968) described heterocystous trichomes from the 800 Ma-old Bitter Springs Formation of Australia. *Anabaenidium johnsonii* has alternatively been interpreted as a preservational artifact (Golubic and Barghoorn, 1977; Gerasimenks and Krylov, 1983). Nagy (1978) reported heterocyst-forming cyanobacteria from the Paleoproterozoic Malmani Dolomite Formation, which later proved to be modern endoliths that penetrated older rocks (Mendelson and Schopf, 1982). Sastry et al. (1972) reported heterocysts from the subsurface Ganga Basin sediments of India. The unequivocal occurrence of Proterozoic heterocysts, demonstrating their unique functional morphology, remains undemonstrated (Wilmotte and Golubic, 1991). Golubic et al. (1995) suggested that the Proterozoic heterocyst-forming cyanobacteria existed, lived in terrestrial and coastal marine environments, and were preserved but remained unrecognized. In spite of these limitations in our understanding of akinetes, based on morphometric and sedimentary behavior comparison with the akinetes of modern bloom-forming *Anabaena*, Golubic et al. (1995) suggested that *Archaeoellipsoides* are fossilized akinetes. Butterfield (2015) also considered that the fossil *Archaeoellipsoides* possibly represents akinetes of Nostocalean cyanobacteria. Nonetheless, at present, the supporting evidence and arguments are less than compelling. To date, the most convincing Stigonematalean heterocysts and associated akinetes have been recorded in terrestrial hot springs known from the early Devonian Rhynie chert (Croft and George, 1959).

### Implications for Paleoenvironmental Settings

The occurrence of authigenic doubly-terminated quartz crystals is usually indicative of a hypersaline environment (Flügel, 2004). The profused development of authigenic doubly-terminated quartz crystals in the Salkhan Limestone suggests the input of silica solution in the depositional milieu in the colloidal form and its subsequent gradual growth as euhedral quartz crystals. Such developments have been noted in various geological settings and ages and are common in evaporitic and Sabkha conditions (Tarr and Lonsdale, 1929; Grimm, 1962; Albright and Lueth, 2003). The source of colloidal silica which played a crucial role in the formation of quartz crystals is not yet fully clear in the context of the Salkhan Limestone; however, precipitation of chert/silica in peritidal carbonate sequences is well established (Knoll, 1985a). The occurrence of radial fan-fabrics in the same chert unit also suggests early diagenetic replacement of aragonitic and gypsum crystals (Figure 9) (Sharma and Sergeev, 2004). Tarr and Lonsdale (1929) suggested the critical role of divalent calcium in gypsum which induces coagulation and precipitation of silica as quartz near the sediment/water interface. Aragonite and gypsum precipitation is common in Sabkha environments, intertidal zones, and lagoonal environments where hypersaline conditions frequently develop over time. The occurrence of different varieties of stromatolites and microfossil assemblages in the Salkhan Limestone was used to interpret their depositional environments; intertidal to supratidal (Kumar, 1978a,b; Sharma, 2006b); intertidal to subtidal (McMenamin et al., 1983); shallow intertidal (Venkatachala et al., 1990; Kumar and Srivastava, 1995); and lagoonal intertidal to shallow marine subtidal environment (Gupta et al., 2003).

Extant *Entophysalis* usually found in the warm shallow hypersaline water bodies of the intertidal zone (Fritsch, 1945; Golubic and Hofmann, 1976) and *Eoentophysalis* dominated the microfossil assemblage recorded at Newari as well as other localities of the Salkhan Limestone, suggesting an intertidal depositional environment (Kumar and Srivastava, 1995; Sharma, 2006a). Radial fan-fabric development after aragonite crystals (Sharma and Sergeev, 2004), silica replacement features characteristics of shallow marine saline water (Sharma, 2006a), and profused occurrence of *Eoentophysalis* (Sharma, 2006a; Shukla and Sharma unpublished data) in the Salkhan Limestone indicate the evaporitic hypersaline depositional environment for the stromatolitic fine laminated chert and doubly-terminated quartz crystal-bearing unit in the Newari and Bargawan localities of the Sonbhadra district. The prevalence of apparently excessive mucilage-encompassed *Eoentophysalis* suggests harsh environmental conditions during the deposition of this unit. The occurrence of akinetes further supports the notion that the depositional conditions were not conducive for normal cyanobacterial growth. Similar depositional conditions have been noted at several places throughout the Proterozoic and are consistent with the Salkhan Limestone shallow hypersaline depositional environment. These places include the Upper Mt. Isa Group, Queensland, Australia (Neudert and Russell, 1981); the Dismal Lakes Group, Canada (Horodyski and Donaldson, 1983); the Sibley Group, Ontario, Canada (Bartley et al., 2015); and Sabkha deposits reviewed by Knoll (1985a,b).

## Conclusion

Study of the stromatolitic chert unit of the Salkhan Limestone (>1600 Ma) exposed at Newari and Bargwan localities reveals the presence of akinetes belonging to the morphogenus *Archaeoellipsoides* and its seven different species, authigenic doubly-terminated quartz crystals, and radial fan-fabrics after the formation of aragonitic crystals have recorded in the petrographic thin sections. The extensive occurrence of mucilage-encompassed *Eoentophysalis* (although not part of the present study) and *Archeaoellipsoides* suggests extreme stress in the depositional environment which triggered mucilage production in certain coccoidal benthic cyanobacteria to overcome extreme conditions such as desiccation and high temperature, leading to akinete production in filamentous cyanobacteria. The occurrence of akinetes can also be used as a proxy to interpret the extremely harsh conditions for the thriving entities in the depositional environment. To this end, the Salkhan Limestone has provided important indicators which can also be used in various geological stages.

## Author Contributions

MS conceived and designed the project, undertook multiple field visits in different localities for collection of samples, placed them in the proper stratigraphic order, prepared and completed the initial scanning of the slides from productive samples for microfossils, and discussed the implications of the data with BS and finalized the manuscript. BS jointly conceived the project, undertook field visit in one of the productive localities for collection of samples, prepared a large set of slides and scanned them for microfossils, generated statistical data, and jointly discussed and prepared the manuscript with MS.

## Conflict of Interest Statement

The authors declare that the research was conducted in the absence of any commercial or financial relationships that could be construed as a potential conflict of interest.

## Supplementary Material

SUPPLEMENTARY DATA Localities and coordinates for all the species of *Archaeoellipsoides* recorded from the Salkhan Limestone.
