# Supplementary material for: Akinetes From Late Paleoproterozoic Salkhan Limestone (>1600 Ma) of India: A Proxy for Understanding Life in Extreme Conditions
Source: Front Microbiol. 2019 Mar 12;10:397. doi: 10.3389/fmicb.2019.00397 (PMC6423410; doi:10.3389/fmicb.2019.00397)
Supplement: SUPPLEMENTARY DATA — Localities and coordinates for all the species of Archaeoellipsoides recorded from the Salkhan Limestone. [file Data_Sheet_1.PDF]

Details for *Archaeoellipsoides* species noted in Fig. 5.

S. No.	Localities (India)	Slide No	Coordinates			Species Name
			EFL	X	Y	
a	Newari, Uttar Pradesh	BSIP-15735	M47/2	47.6	113.8	*A. elongatus*
b	Newari, Uttar Pradesh	BSIP-10907	N41/4	35.8	113.2	*A. dolichos*
c	Jata Shankar Pahari, Uttar Pradesh	BSIP-15729	D31/4	31.7	121.1	*A. bactroformis*
d	Newari, Uttar Pradesh	BSIP-15733	R43/3	43.3	108.7	*A. dolichos*
e	Newari, Uttar Pradesh	BSIP-15735	L30/3	30.4	114.4	*A. bactroformis*
f	Newari, Uttar Pradesh	BSIP-15736	K51/2	51.4	115.10	*A. elongatus*
g	Newari, Uttar Pradesh	BSIP-15734	N48/4	48.9	112.6	*A. major*
h	Newari, Uttar Pradesh	BSIP-15733	R47/4	47.10	108.7	*A. minor*
i	Nauhatta, Bihar	BSIP-14994	L35/2	35.8	115.1	*A. conjuctivus*
j	Jata Shankar Pahari, Uttar Pradesh	BSIP-15107	P35/3	35.4	110.6	*A. grandis*
k	Jata Shankar Pahari, Uttar Pradesh	BSIP-15731	L51/2	51.1	115.1	*A. grandis*
l	Jata Shankar Pahari, Uttar Pradesh	BSIP-15728	T45/2	45.6	107.1	*A. bactroformis*
m	Jata Shankar Pahari, Uttar Pradesh	BSIP-15728	T45/2	45.6	107.1	*A. major*
n	Jata Shankar Pahari, Uttar Pradesh	BSIP-15730	M38/4	38.9	113.7	*A. grandis*
o	Jata Shankar Pahari, Uttar Pradesh	BSIP-15741	E 46/4	46.6	120.2	*A. conjuctivus*
p	Nauhatta, Bihar	BSIP-15727	P36/3	36.4	110.5	*A. major*
q	Nauhatta, Bihar	BSIP-14994	J42/3	42.1	116.7	*A. grandis*
r	Nauhatta, Bihar	BSIP-14999	G31/1	31.6	119.2	*A. major*
s	Jata Shankar Pahari, Uttar Pradesh	BSIP-15741	E 46/2	46.3	120.5	*A. grandis*

Details for *Archaeoellipsoides* species noted in Fig. 6.

S. No.	Localities (India)	Slide No	Coordinates			Species Name
			EFL	X	Y	
a	Newari, Uttar Pradesh	BSIP-15723	J28/4	29.5	117.1	*A. minor*
b	Newari, Uttar Pradesh	BSIP-15723	J29/1			
c	Newari, Uttar Pradesh	BSIP-15735	N41/4	41.6	112.5	*A. major*
d	Newari, Uttar Pradesh	BSIP-15735	L25/4	26.4	114.5	*A. bactroformis*
e	Newari, Uttar Pradesh	BSIP-15734	R33/1	33.2	109.1	*A. minor*
f	Newari, Uttar Pradesh	BSIP-15724	O36/4	36.8	111.4	*A. bactroformis*
g	Nauhatta, Bihar	BSIP-14993	K59/2	59.3	116.1	*A. minor*
h	Nauhatta, Bihar	BSIP-14997	O39/4	39.8	111.5	*A. bactroformis*
i	Newari, Uttar Pradesh	BSIP-15725	W37/3	37.1	104.8	
j	Nauhatta, Bihar	BSIP-14998	H29/4	30.2	117.4	
k	Nauhatta, Bihar	BSIP-14999	M52/2	52.3	113.10	*A. major*
l	Newari, Uttar Pradesh	BSIP-15734	M45/4	45.5	113.4	*A. bactroformis*

Details for *Archaeoellipsoides* species noted in Fig. 7.

S. No.	Localities (India)	Slide No	Coordinates			Species Name
			EFL	X	Y	
a	Bargwan, Uttar Pradesh	BSIP-15732	Z45/2	45.7	101.1	*A. minor.*
b	Newari, Uttar Pradesh	BSIP-15740	N28/3	28.5	112.6	
c	Newari, Uttar Pradesh	BSIP-15737	V37/2	37.10	105.2	
d	Newari, Uttar Pradesh	BSIP-15737	N39/2	39.7	113.1	
e	Newari, Uttar Pradesh	BSIP-15726	L34/2	37.1	114.6	
f	Newari, Uttar Pradesh	BSIP-15734	R37/4	37.5	108.10	
g	Nauhatta, Bihar	BSIP-14998	J32/2	33.1	116.9	
h	Newari, Uttar Pradesh	BSIP-15738	M38/1	38.1	114.7	*A.conjuctivus*
i	Newari, Uttar Pradesh	BSIP-15738	M38/1			
j	Newari, Uttar Pradesh	BSIP-15739	N27/2	28.1	113.5	*A.elongatus*
